# A Taxonomy and Results from a Comprehensive Review of 28 Maternal Health Voucher Programmes

**Published:** 2013-12

**Authors:** Ben W. Bellows, Claudia M. Conlon, Elizabeth S. Higgs, John W. Townsend, Matta G. Nahed, Karen Cavanaugh, Corinne G. Grainger, Jerry Okal, Anna C. Gorter

**Affiliations:** ^1^Population Council, Nairobi, Kenya; ^2^United States Agency for International Development, Washington, DC, USA; ^3^National Institute of Allergy and Infectious Diseases, Bethesda, MD, USA; ^4^Options Consultancy Services Ltd., London, UK; ^5^Instituto Centro-Americano de la Salud, Nicaragua

**Keywords:** Demand-side financing, Maternal healthcare, Results-based financing, Vouchers

## Abstract

It is increasingly clear that Millennium Development Goal 4 and 5 will not be achieved in many low- and middle-income countries with the weakest gains among the poor. Recognizing that there are large inequalities in reproductive health outcomes, the post-2015 agenda on universal health coverage will likely generate strategies that target resources where maternal and newborn deaths are the highest. In 2012, the United States Agency for International Development convened an Evidence Summit to review the knowledge and gaps on the utilization of financial incentives to enhance the quality and uptake of maternal healthcare. The goal was to provide donors and governments of the low- and middle-income countries with evidence-informed recommendations on practice, policy, and strategies regarding the use of financial incentives, including vouchers, to enhance the demand and supply of maternal health services. The findings in this paper are intended to guide governments interested in maternal health voucher programmes with recommendations for sustainable implementation and impact. The Evidence Summit undertook a systematic review of five financing strategies. This paper presents the methods and findings for vouchers, building on a taxonomy to catalogue knowledge about voucher programme design and functionality. More than 120 characteristics under five major categories were identified: programme principles (objectives and financing); governance and management; benefits package and beneficiary targeting; providers (contracting and service pricing); and implementation arrangements (marketing, claims processing, and monitoring and evaluation). Among the 28 identified maternal health voucher programmes, common characteristics included: a stated objective to increase the use of services among the means-tested poor; contracted-out programme management; contracting either exclusively private facilities or a mix of public and private providers; prioritizing community-based distribution of vouchers; and tracking individual claims for performance purposes. Maternal voucher programmes differed on whether contracted providers were given training on clinical or administrative issues; whether some form of service verification was undertaken at facility or community-level; and the relative size of programme management costs in the overall programme budget. Evidence suggests voucher programmes can serve populations with national-level impact. Reaching scale depends on whether the voucher programme can: (i) keep management costs low, (ii) induce a large demand-side response among the bottom two quintiles, and (iii) achieve a quality of care that translates a greater number of facility-based deliveries into a reduction in maternal morbidity and mortality.

## INTRODUCTION

It is increasingly clear that Millennium Development Goal 4 and 5 will not be achieved in many low-income countries (LMICs). According to a recent analysis, at the current rate of change in the neonatal mortality rate (NMR) and maternal mortality ratio (MMR), only 31 countries will meet MDG 4 and still fewer (thirteen) will achieve MDG 5 (1). In many of these countries, the burden of disease and barriers to high-quality healthcare fall disproportionately on lower wealth quintiles (2). Due to these slow improvements in health and the large gaps in equity, there have been calls for global stakeholders to pledge or renew commitments for greater resources and to prioritize strategies that target populations and geographic locations where MMR and NMR are the highest. In addition, there is growing awareness that fostering equitable access to health systems and higher-quality services will figure as significant components in the post-2015 agenda on universal health coverage (3).

**Figure 1.**
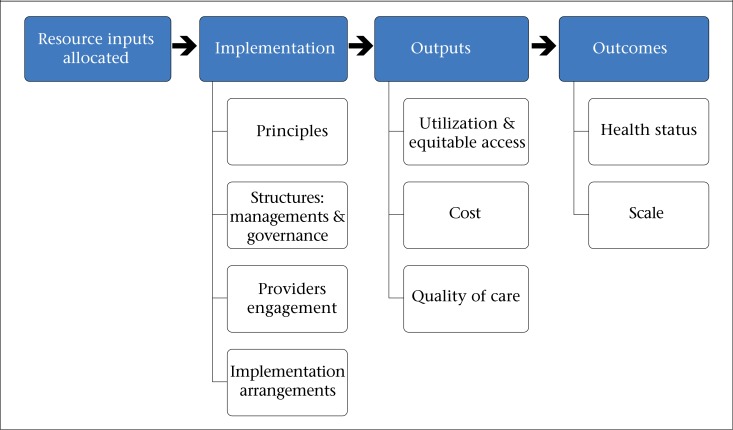
Taxonomy to catalogue designs, functions, and results of reproductive health voucher programmes

The United States Agency for International Development (USAID) held an Evidence Summit in April 2012 to assess the evidence on financial incentives to enhance the provision and use of maternal health services. Financial incentives can be used for accelerating increased coverage, quality, and the use of prenatal and postnatal services and for fostering healthy behaviours during pregnancy and the neonatal period (4-6). A commonly-accepted umbrella term for these approaches is results-based financing (RBF). Related terms include performance-based incentives (PBIs), output-based aid (OBA), and pay-for-performance (P4P) (7). RBF programmes can be characterized by where they place incentives, targeted to either the demand-side or supply-side while acknowledging that demand and supply are intrinsically linked. For the purposes of classification, each type of intervention is labelled according to its unique features that operate on either demand or supply.

In demand-side healthcare financing programmes, the objective is to remove barriers to healthcare-seeking behaviours through economic subsidies. These schemes range from universal with a ‘broad’ benefits package (e.g. national social insurance) to those targeted with a ‘narrow’ benefits package (e.g. maternal health voucher programmes). Although removing barriers to the use of services by clients is a central objective, there are anticipated supply-side effects. Healthcare providers are often paid according to the number of clients treated (output-based) or a clearly-defined performance achievement (quality-adjusted output payments). A key feature then is the direct link between the subsidy to the intended beneficiary on the demand side and the desired output on the supply side. In many of these programmes, the focus on demand is justified by historical, persistent unmet needs for specific services [e.g. treatment for sexually transmitted infections (STIs) for socially-marginalized groups or products (e.g. insecticide-treated bednets or contraceptives (8,9).

Given the high levels of inequality observed in maternal and neonatal health outcomes and the use of healthcare, targeted demand-side strategies have the potential to stimulate the use of public health goods and services among the low-income segment of the market where uptake of high-quality health services is often the weakest (2). Vouchers targeted at underserved populations are increasingly used in promoting priority health services and are redeemable for a defined service package at accredited health facilities. Most voucher programmes to date have been designed to increase access to maternal, sexual and reproductive health (SRH) services for those who, in the absence of the voucher, would not have sought care.

To provide guidance to donors and governments who have an interest in launching or expanding reproductive health voucher programmes, this paper presents a taxonomy of knowledge about programme implementation and impact (Figure 1), using a “results chain” to frame the review (10).

Two recent reviews have broadly summarized the literature drawing from studies of family planning vouchers, maternal health vouchers, and other reproductive and sexual health vouchers. Both reviews found robust evidence that voucher programmes can increase utilization of health services and showed modest evidence that voucher programmes both improve the quality of service provision and target the resources effectively to specific populations (11,12). There was limited evidence available to determine whether vouchers affect health status or technical efficiency. However, in both Uganda and Nicaragua, a reduction was found in the prevalence of several STIs associated with launching of the voucher programme (13,14).

This current review of maternal health voucher programmes goes beyond the prior reviews to catalogue characteristics and identify essential characteristics of sustainable successful maternal health voucher programmes. It includes a thorough assessment of 28 maternal voucher programmes identified in a comprehensive review of 40 reproductive health voucher programmes; these were cross-checked against programmes identified in an unpublished report of the 2012 USAID Evidence Summit on Maternal Health Finance (15,16). The 28 voucher programmes were examined in five key categories: (i) general design principles; (ii) governance and management; (iii) benefits package and beneficiary targeting; (iv) provider and reimbursement policies; and (v) implementation issues, such as marketing, training, voucher distribution, claims processing, and mechanisms for monitoring, evaluation, and fraud control (17-24). To date, the knowledge of programme design and function—evidence on how vouchers are implemented—has been largely uncategorized. The challenge with organizing such knowledge is that it goes unpublished, often located in programme reports or known only to programme implementers. This paper presents a taxonomy of voucher implementation to organize what is known about maternal health voucher programmes with the goal of identifying a future research and policy agenda to contribute to further understanding of the following policy-relevant questions:

1. Can a voucher programme operate on low management costs and direct the bulk of the funds and services to the bottom two wealth quintiles?
2. If programmes can cost-effectively serve the poor, do clients respond? Was a financial barrier the primary reason for non-use of services before the voucher programme?
3. What is the cost per unit of increase in facility-based deliveries among the bottom two wealth quintiles?
4. In a causal model, what is the estimated reduction in maternal mortality with the observed increase in facility-based deliveries?

## MATERIALS AND METHODS

This review draws on a subset of voucher programmes offering maternal health voucher services identified in a larger dataset of reproductive health voucher programmes described in detail by Grainger *et al*. (16). The review by Grainger *et al*., focusing on programme design and function (not scientific studies), was conducted in three steps to screen published and grey literature. First, the methods and results of two prior literature reviews were compared to identify the voucher programmes in published studies. As voucher programmes were identified, the name of the programme and other relevant information were confirmed through supplemental searches. A detailed description of the search process is described by Grainger *et al*. (16).

The second step in this review involved consulting experts and contacts to identify any new programmes and further programme references, drawing from reports, operational manuals, newsletters, and other relevant documents.

Criteria for inclusion and exclusion of voucher programmes followed those used by Grainger and colleagues (16):

- Inclusion of voucher programmes which do not use a physical voucher but function in all other respects as a voucher programme (e.g. targeting the poor through the use of Below Poverty Line or BPL cards in India);
- Exclusion of programmes that use vouchers for goods (condoms, insecticide-treated bednets) opposed to services. Structural and implementation arrangements differ considerably between vouchers for goods and those for services;
- Exclusion of those voucher programmes that are operating in high-income countries;
- Exclusion of programmes where there is no reimbursement to the provider, such as programmes where a voucher is used as a marketing tool, for referral services between health facilities only, or for research (tracking of clients, etc.). It also excludes programmes where vouchers are given to women in exchange for a conditional cash transfer (CCT) with no provider payment;
- Exclusion of voucher programmes that started distribution of vouchers after June 2011.

For the final methodological step, information was extracted on each programme and fed into a dataset that captured more than 120 programme characteristics belonging to one of the following five major categories relating to design and function of the programmes:

1. General principles: Programme objectives, size, and coverage, financing, and timeframe;
2. Governance and management (structural elements): policy environment, regulatory framework, managing agency, and its relation to contracted providers;
3. Benefits package and beneficiary targeting: Benefit and client policies, such as services covered, distribution strategies (i.e. sold or freely distri-buted), and targeting approaches;
4. Providers: Type of providers, presence of competition between facilities, selection and contracting, price of services, and reimbursement to providers;
5. Implementation arrangements: Marketing, training, voucher distribution and sales, claims processing; mechanisms for monitoring and evaluation, and fraud control.

## RESULTS

### General principles: objectives, timeframe, and financing

**Figure 2.**
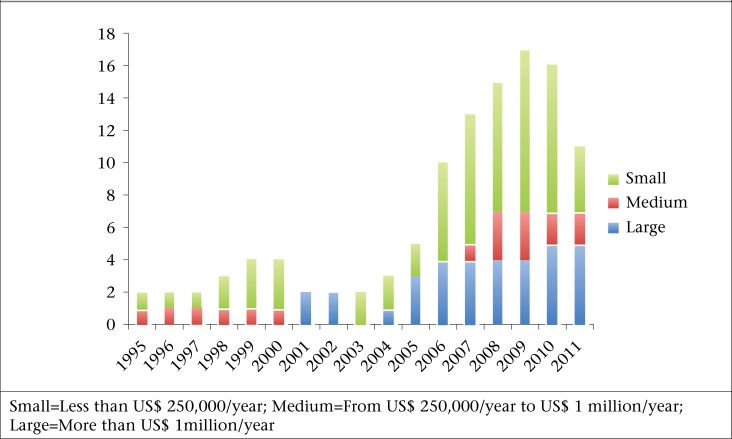
Number of active maternal health voucher programmes in low- and middle-income countries year-by-year

In total, 28 safe motherhood (SM) voucher programmes operating in 10 countries were identified. Most programmes had the stated objective to improve the use of maternal health services, particularly among the poor while a few programmes emphasized the importance of improving population health and reducing the burden of out-of-pocket spending on maternal health services. Figure 2 details the number of active SM voucher programmes operating each year from 1995 to 2011. As seen in the Figure, SM voucher programmes started in 1995 with one small and one medium programme and continued in this modest fashion until 2006 when a substantial growth occurred in SM voucher programmes. The majority of the programmes (64%) are small, defined as those with an annual budget of US$ 250,000 or less.

Table 1 provides an overview of information for the 28 SM voucher programmes identified in this analysis. Twenty-four were located in Asia (Armenia, Bangladesh, Cambodia, China, India, Indonesia, and Pakistan) and four in Africa (one each in Kenya and Sierra Leone, and two programmes in Uganda). Many countries had more than one SM voucher programme, with India having the highest number of eight programmes located in different cities and states. Funders, including external aid actors, non-governmental organizations (NGOs), social franchising organizations, private for-profit organizations, and research organizations, set up most programmes while six were government-led. Most of the programmes (61%) were stand-alone SM voucher programmes while the others also had a family planning and/or STI component to the voucher programme. More than half of the SM voucher programmes were described as ‘pilots’ to be potentially expanded if successful.

### Governance and management

The three main participants in any health voucher programme are the service providers, the clients (or patients), and the voucher management agency (VMA). In the implementation data, three variations on this model were evident. There were six government-run programmes in South Asia with a focus on improving the operational efficiency and equitable access within the public health system. In these contexts, the voucher essentially functions as a waiver system for poor clients to avoid user fees and informal charges while acting as an incentive for improved care on the provider side. In some of these six government-run initiatives, voucher clients also received a cash payment conditional on delivering at an approved facility.

Six social franchise (SF) programmes also managed voucher initiatives to generate demand and subsidize access to services at franchised facilities. In social franchises, private healthcare providers are contracted under a common brand to provide socially-beneficial, typically outpatient services to increase delivery of health services (25,26). One of the largest social franchises is Greenstar in Pakistan, which began as a pilot maternity voucher scheme in Charsadda and Jhang districts to reach poor clients in 2010 (27,28). Although most of these franchises only contracted private facilities, one SF programme in Bangladesh, run as a partial franchise, contracted services from public, private-for-profit and private-not-for-profit facilities (29).

In the third variation, NGOs or private firms managed 16 voucher programmes on a contractual basis for donors or the local government. This management structure, in which the donor or government procured services from external non-state agencies, provided valuable alternative experiences to the long-dominant, input-based financing models.

### Benefits package and beneficiary targeting

The voucher benefits packages and the criteria for who would qualify varied among programmes. All 28 programmes either contracted directly within the network or coordinated access to out-of-network third party providers for antenatal care, delivery, and postnatal care. Nearly all programmes paid for normal delivery with basic emergency obstetric care (26 of 28 programmes). One programme referred deliveries to the public sector, and one programme lacked sufficient information to determine whether deliveries were included in the programme budget or were referred to external public-sector providers.

According to WHO guidelines, comprehensive emergency obstetric care includes provision of surgery and blood transfusions in addition to the seven signal functions of basic emergency obstetric care (30). Among the 28 maternal voucher programmes, 21 contracted comprehensive emergency obstetric care from a subset of their accredited providers. Six programmes lacked this capacity among their accredited providers and instead referred complicated cases to external facilities, usually in the public sector. One programme lacked sufficient information to determine whether the programme contracted providers with capacity to provide comprehensive emergency obstetric care.

Of the 28 voucher programmes, 10 offered transport to facilities from the client's home; two covered transport to another facility for referral; 13 programmes did not offer any transport subsidy; and three programmes had no information.

**Figure 3.**
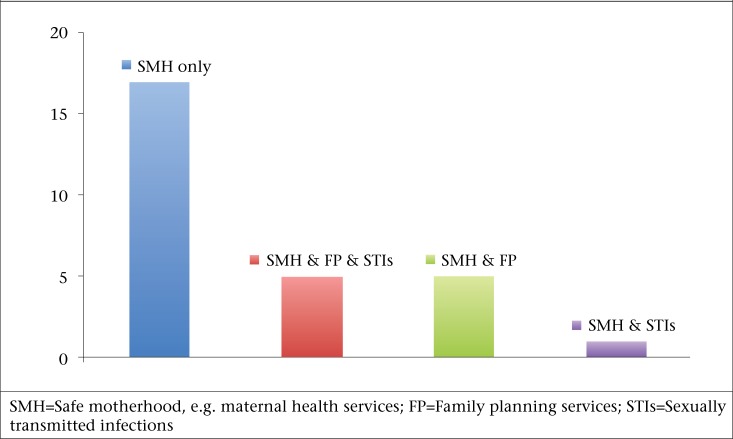
Twenty-eight maternal voucher programmes in low- and middle-income countries with additional services

In addition to the core maternal health package, 10 programmes also offered a separate family planning (FP) voucher service, and six programmes offered a voucher for treatment of STIs. In five of these 10 programmes, both STI treatment and FP services were offered as separate vouchers. The remaining 17 voucher programmes in this review offered only maternal health services (Figure 3).

**Table 1.** Overview of the 28 maternal health voucher programmes in low- and middle-income countries

Programme	Years	Primary funders	Programmme-size	Objective
1. Armenia	2008-2012	Government	Large	Reduce OOP
2. Bangladesh (A)	2004-2012	Government, multidonor	Large	Improve health status
3. Bangladesh (B)	2006-2008	DFID, other donors	Small	Increase healthcare-use
4. Bangladesh (C)	2007-2008	UNFPA	Small	Increase healthcare-use
5. Bangladesh (D)	2006-2011	EC	Small	Increase use among poor
6. Cambodia (A)	2007-2010	BTC	Small	Increase healthcare-use
7. Cambodia (B)	2008-2010	UNFPA	Small	Increase use among poor
8. Cambodia (C)	2009-2012	USAID	Small	Increase use among poor
9. Cambodia (D)	2010-2013	KfW	Large	Increase use among poor
10. China (A)	1995-2001	World Bank	Small	Improve health status
11. China (B)	1998-2007	World Bank, DFID, Government	Small	Increase use among poor
12. India (A)	2006-2011	USAID	Small	Improve health status
13. India (B)	2008-2012	USAID	Medium	Improve health status
14. India (C)	2007-2009	USAID, Government	Small	Increase use among poor
15. India (D)	2005-2012	Government	Large	Improve health status
16. India (E)	2007-2012	USAID, Government	Medium	Increase use among poor
17. India (F)	2008-2010	BMGF	Medium	Increase use among poor
18. India (G)	1999-2003	Government	Small	Improve health status
19. India (H)	2003-2012	Government	Small	Increase use among poor
20. Indonesia	1998-2004	World Bank	Medium	Increase use among poor
21. Kenya	2005-2012	KfW, Government	Large	Increase use among poor
22. Pakistan (A)	2008-2009	USAID	Small	Increase use among poor
23. Pakistan (B)	2009-2012	PSI, Government	Small	Increase use among poor
24. Pakistan (C)	2010-2011	KfW	Small	Increase use among poor
25. Pakistan (D)	2009-2012	Government	Small	Increase use among poor
26. Sierra Leone	2009-2012	MSI	Small	Improve health status
27. Uganda (A)	2006-2012	KfW, World Bank (GPOBA), DFID	Large	Improve health status
28. Uganda (B)	2009-2012	BMGF, Futures Health Systems Consortium	Small	Increase healthcare-use

BMGF=Bill & Melinda Gates Foundation

BTC=Belgium Technical Cooperation

DFID=Department for International Development, UK

EC=European Community

GPOBA=Global Partnership on Output-based Aid

KfW=German Development Bank

MSI=Marie Stopes International

OOP=Out-of-pocket spending on healthcare

PSI=Population Services International

UNFPA=United Nations Population Fund

USAID=United States Agency for International Development

Use among poor=Increase use of voucher-related, subsidized health services among the poor as defined by that voucher programme

Small size=<US$ 250,000/year

Medium size=From US$ 250,000/year up to US$ 1 million/year

Large size=>US$ 1 million/year

Details on the type of beneficiary targeting were available for 26 of the 28 maternal health voucher programmes. Among the 26, half of the programmes targeted low-income women, using a poverty assessment tool that measured household wealth, often based on a combination of household assets and consumption measures. Five programmes used geographic targeting where women were eligible based on their residence in a well-defined low-income area. Another six programmes used a mix of geographic and means-testing for targeting. Details on the implementation of the SM voucher programmes are listed in Table 2.

### Providers

When identifying health service providers, SM voucher programmes can (in theory) choose to contract with public providers, private providers, or a mix of both. In reality, the national political economy, the policy environment, and the functional state of health facilities can drive decisions regarding how and with whom to contract. Of the 28 programmes, 61% contracted exclusively or mostly with private healthcare providers while 21% contracted mostly or exclusively with public healthcare providers; another 18% contracted a mix of both public and private providers. When contracting providers, some programmes deliver clinical training to emphasize quality in service delivery (particularly those originated by social franchises), and some provide administrative training on how voucher claims should be filed. Clinical training of providers occurred in 43% of the programmes, and administrative training was conducted for 39% of programmes. One-quarter (7 programmes) offered both clinical and administrative training.

### Implementation arrangements

SM voucher implementation involves more functions than identifying the beneficiary and contracting the provider. There are processes needed to reach the client, including accreditation and branding of service sites, whether and what to charge for the voucher, marketing strategies to generate demand, and voucher distribution schemes. Five programmes sold SM vouchers to beneficiaries for a nominal amount equivalent to US$ 0.75 to 2.00 while 20 programmes distributed the voucher at no cost to consumers. Three programmes had no documentation on whether the voucher was free to consumers or not.

**Table 2.** Programme characteristics of 28 maternal voucher initiatives in low- and middle-income countries

Programme	Providers used	Training of providers	Targeting (beneficiary identified)	Voucher price to client	Marketing	Voucher management agency	Voucher package	Verify service delivery?
1. Armenia	Mostly private	None	No targeting	Free	MM, HV	Government	SM, Child health	Yes
2. Bangladesh (A)	Mostly public	Admin.	Mixed	Free	CM, HV	Government	SM, Transport	Yes
3. Bangladesh (B)	Private	None	Mixed	Free	None	Research institute	SM	Yes
4. Bangladesh (C)	Mixed	Clinical	Means-test	Free	CM, HV	NGO	SM, Transport	No
5. Bangladesh (D)	Mixed	Clinical	Means-test	Free	CM, HV	NGO (SF)	SM, Child health, Transport	No
6. Cambodia (A)	Public	None	Means-test	Free	MM, CM, HV	NGO	SM, Transport	Yes
7. Cambodia (B)	Public	Admin. & clinical	Means-test	Free	HV	NGO	SM, FP, Post-abortion, STI, PMTCT, Transport for referrals	Yes
8. Cambodia (C)	Public	None	No targeting	Free	None	NGO	SM, HIV VCT	No
9. Cambodia (D)	Mixed	Admin.	Mixed	Free	MM, CM, HV	NGO/Private for-profit	SM, FP, Abortion, Transport	Yes
10. China (A)	Public	None	Means-test	Free	None	Government	SM, Child health, Transport for referrals	No
11. China (B)	Public	None	Means-test	Free	None	Government	SM, Child health, Hypertension, STIs	No
12. India (A)	Private	None	Geographic	Free	MM, HV	NGO	SM, Transport	No
13. India (B)	Private	None	Means-test	Free	MM, CM, HV	Government	SM	Yes
14. India (C)	Private	Admin. & clinical	Means-test	Free	CM, HV	Government (parastatal)	SM, FP, STI	Yes
15. India (D)	Private	None	Means-test	Free	CM, HV	Government	SM, Transport	No
16. India (E)	Private	Clinical	Means-test	Free	CM, HV	NGO	SM, FP	No
17. India (F)	Private	None	Geographic	Free	HV	NGO	SM, FP, STI	No
18. India (G)	Private	Clinical	Geographic	Free	CM, HV	NGO	SMH, FP, STI, CH, OP, Transport	No
19. India (H)	Private	None	Geographic	Dnk	CM, HV	NGO	SM, Transport	No
20. Indonesia	Private	Admin. & clinical	Mixed	Free	CM, HV	Government	SM, FP	No
21. Kenya	Mixed	Admin.	Means-test	US$ 1.50	MM, CM	Private for-profit	SM, FP, GBV	Yes
22. Pakistan (A)	Private	Admin. & clinical	Means-test	US$ 1.20	HV	NGO (SF)	SM only	Yes
23. Pakistan (B)	Mostly private	Admin. & clinical	Means-test	US$ 1.20	HV	NGO (SF)	SM only	Yes
24. Pakistan (C)	Mostly private	Admin. & clinical	Unknown	Dnk	None	NGO (SF)	SM, Transport	No
25. Pakistan (D)	Mostly private	None	Unknown	Dnk	None	Private for-profit	SM	No
26. Sierra Leone	Mostly private	Admin.	Mixed	US$ 0.75	CM, HV	NGO (SF)	SM, FP	No
27. Uganda (A)	Private	Admin. & clinical	Mixed	US$ 1.10	MM, CM, HV	NGO (SF)	SM, FP, STI	Yes
28. Uganda (B)	Mixed	Clinical	Geographic	Free	MM, CM, HV	Research institute	SM, Transport	No

CH=Child health services, including vaccinations

CM=Community meetings

Dnk=Do not know

FP=Family planning

HV=Home visit

GBV=Gender-based violence recovery services

MM=Mass media

NGO=Non-governmental organization

OP=outpatient care

SF=Social franchise

SM=Safe motherhood

STI=Sexually transmitted infections

Almost all SM voucher programmes used some form of marketing, at least at the start of the programme. The most common form of marketing was home visits linked to voucher distribution (75%), followed by community meetings (57%) and mass media message (29%). Most programmes had community health workers and community volunteers distributing vouchers in the community, away from facilities, to identify new clients and generate demand for facility services. In two programmes, the staff at the participating facilities distributed the vouchers, which raised questions about how effectively the programme was reaching users who would not otherwise have sought facility-based care.

Strikingly, information about management or administrative costs was missing for 21 of the 28 maternal voucher programmes. Among the seven programmes with data on costs, two spent less than 10% of the overall programme budget on programme management, four programmes spent 20-30% on programme management, and one programme used 50% of the overall budget on management costs. These figures can be difficult to interpret without knowing more about what they contain and what period of time they refer to, since higher costs are associated with programme start-up or where the risks of mismanagement of funds are perceived to be higher. There are differing definitions and internal reporting methods, different management activities undertaken by particular VMAs, various programme designs, and varying country contexts. To further complicate any comparisons, lower administrative costs do not necessarily produce optimal administration; it is equally important to consider the outcomes achieved by the voucher programme (Table 3).

In spite of the limitations, the ratio of administrative costs to service delivery costs does provide a measure of efficiency, particularly if tracked over time and can be used for comparing similarly-designed programmes. The literature on administrative costs in health insurance notes that public-sector management is generally quite a small percentage of the total programme cost (under 6%) and higher for private health insurance (9-17%) (31,32). Voucher programmes—once they have scaled to several hundreds of thousands of billable claims per year—should expect to see comparable administrative cost levels.

One important task of VMAs is to verify actual service delivery to the target populations. This is often done through a review of claims to confirm that the information is accurate and valid. Other verification means include facility-based exit-interviews or community-based surveys of clients. Verification occurs at one or more of the three points in the service delivery chain: (i) the community distributor may authenticate the poverty status of the beneficiary and unique number on the voucher, (ii) at the facility, the provider may authenticate the voucher at client registration, and (iii) at the claims processing stage, the details on the claim are routinely vetted for medical plausibility and proper recording. Only 12 of the 28 SM voucher programmes had some form of service-delivery verification process in place. The data did not indicate what type of verification activity was most common.

**Table 3.** Improvements in coverage among 11 maternal health voucher programmes in 15 voucher studies

Programme/Study no.	Country	Scheme(s)	Study	Sample-size	Baseline indicator (prior to vouchers or among control group)	Effect identified by study among beneficiaries/at follow-up†
1.1	Bangladesh	Vouchers for ANC, delivery, and PNC in districts with individual poverty targets and districts with universal targeting. Programme popularly known as the demand-side financing (DSF) voucher. Government directly managed the programme	Cross-sectional study to measure differences in prevalence after two years of the demand-side financing (DSF) voucher programme (49)	Controls: 1,104 Universal: 552 Means-tested: 552	Among control respondents: - 33.6% use at least 3 ANC - 27.1% had deliveries attended by qualified skilled provider - 18.7% had facility-based deliveries - 20.7% had at least one PNC visit - 9.1% had a C-section	Averaged among ‘universal’ and ‘means tested’ respondents: - 54.8% use at least 3 ANC visits (p<0.001) - 63.7% had deliveries attended by skilled providers (p<0.001) - 37.5% had facility-based deliveries (p<0.001) - 35.6% had at least one PNC visit (p<0.001) - 10.4% had a C-section
1.2	Bangladesh	Vouchers for ANC, delivery, and PNC in districts with individual poverty targets and districts with universal targeting. Programme popularly known as the demand-side financing (DSF) voucher. Government directly managed the programme	Cross-sectional design comparing group means over past six months and comparing service utilization by level of poverty, e.g. concentration indices (50)	600 voucher respondents; 3,000 control respondents	Absolute percentages among control and voucher clients were not reported	At endline, voucher recipients in the project area were more likely to seek care compared to pregnant women not in the programme. Specifically: - 3.6 times more likely to be assisted during delivery (SE 0.14) - 2.5 times more likely to deliver the baby in a health facility (SE 0.182) - 2.8 times more likely to receive postnatal care (SE 0.136) - 2.0 times more likely to seek 3+ antenatal care visits (SE 0.126) - 1.5 times more likely to seek treatment for obstetric complications (SE 0.149) Voucher recipients in bottom tertile were: - 4.3 times more likely to deliver in a health facility - 2 times more likely to use skilled health personnel at delivery than the non-poor recipients
1.3	Bangladesh	Vouchers for ANC, delivery, and PNC in districts with individual poverty targets and districts with universal targeting. Programme popularly known as the demand-side financing (DSF) voucher. Government directly managed the programme	1. Unadjusted cross-sectional estimates of maternal service- use, OOP spending 2. Retrospective birth history difference-in-differences analysis of means by location of ANC and birth, type of birth, and related utilization outcomes (51)	2,208 women were interviewed about most recent birth 2,861 births before and after launching of the programme	Comparison group had - 75.6% attending any ANC - 33.6% at least 3 ANC - 54.9% at least 1 ANC with qualified provider - 27.1% births attended by a qualified provider - 18.7% institutional delivery - 9.1% C-section - 15.0% at least 1 PNC with qualified provider - 89.6% incurred any OOP payment for maternal health	Intervention group had: - 91.6% attending any ANC - 54.8% at least 3 ANC - 79.2% at least 1 ANC with qualified provider - 63.7% birth attended by a qualified provider - 37.5% institutional delivery - 10.4% C-section - 30.4% at least 1 PNC with qualified provider - 86.9% incurred any OOP payment for maternal health Compared to births in non-voucher subdistricts before and after voucher launching, births in voucher subdistricts have, on average, 18.2 percentage points (SE 0.063) higher probability of taking place at facility as reported by fixed effects Model 1 results
2.4	Bangladesh	Vouchers for ANC, delivery, and PNC in districts with individual poverty targets and a third-party voucher management agency (VMA); run as a demonstration pilot	A before-after design with no controls, measuring the use of ANC, delivery, and PNC services (52)	436 at baseline; 414 at endline	Of the baseline group: - 79.3% had at least 1 ANC visit - 58.7% had at least 2 ANC visits - 30.2% had at least 3 ANC visits - 64.8% said money is barrier to ANC - 5.5% had skilled birth attendant - 2.3 delivered at health facility - 45.2% received PNC	Of the endline group: - 89.1% had at least 1 ANC visit - 76.2% had at least 2 ANC visits - 63.4% had at least 3 ANC visits - 0% said money is barrier to ANC - 21.6% had skilled birth attendant - 18.3% delivered at health facility - 60.1% received PNC
3.5	Cambodia	Three operational districts received poverty-targeted vouchers and Health Equity Funds (HEFs) to subsidize access to maternity services for the poor. In addition, performance-based contracts (PBC) to facilities and US$ 12.5/US$ 15 output payments to midwives for facility referrals introduced simultaneous effects on facility deliveries. Intervention in this study was combination of vouchers and HEF	An ecological design using routine health management information system (HMIS) data, voucher utilization data, and comparing year-on-year trends. Supplemented by qualitative methods (53)	N=2,725 vouchers in distribution	Baseline (2006): - 16.4% of all estimated deliveries at facility in voucher + ODs - 24.5% of all estimated deliveries at facility in PBC + ODs - 4.5% of all estimated deliveries at facility in control ODs	Endline (2008): - 28.6% ppt increased to 44.9% deliveries at facility in voucher + ODs - 14.5% ppt increased to 39% deliveries at facility in PBC + ODs - 8.6% ppt increased to 13.1% deliveries at facility in control ODs
4.6	China	Vouchers paid for ANC, delivery, PNC, and treatment for diarrhoea and pneumonia among children below three years of age in Yunnan province	A before-after design with controls comparing change in mean values (K Du. Personal communication, 2001)	Not available	Baseline use of childhood diarrhoea treatment in intervention area 1: - 67.3% among very poor - 75.5% among poor - 77.2% among non-poor Use of childhood diarrhoea treatment in intervention area 2: - 69.5% among very poor - 76.6% among poor - 78.4% among non-poor Use of childhood diarrhoea treatment in control area: - 63.1% among very poor - 72.2% among poor - 75% among non-poor	Endline use of childhood diarrhoea treatment in intervention area 1: - 13.8 ppt increased to 81.1% among very poor - 3.9 ppt increased to 78.5% among poor - 6.9 ppt increased to 82.5% among non-poor Use of childhood diarrhoea treatment in intervention area 2: - 18.5 ppt increased to 82.3% among very poor - 3.8 ppt increased to 79.5% among poor - 6.8 ppt increased to 83.7% among non-poor Use of childhood diarrhoea treatment in control area: - 1.7 ppt increased to 64.1% among very poor - 1.9 ppt increased to 73.6% among poor - 3.2 ppt increased to 77.4% among non-poor
5.7	India	The *Chiranjeevi Yojana* provides free delivery care to women below poverty line (BPL) by private obstetricians. Government pays fixed amount (US$ 4,487) for 100 deliveries, including normal and complicated deliveries. The package also includes free food and postnatal medicines and reimbursement of transport cost (US$ 1.25) for accompanying family members	Cross-sectional analysis of place of last delivery between *Chiranjeevi* beneficiaries (CB) and non-*Chiranjeevi* mothers (NCM) (54)	394 controls; 262 *Chiranjeevi* beneficiaries	Deliveries by controls were: - 21% at home - 77% at private facilities - 2.6% at public facilities	Deliveries by *Chiranjeevi* beneficiaries were: - 0.4% at home - 97% at private facilities - 2.0% at public facilities Significance not reported for these findings
6.8	India	Delhi local government contracted 36 private hospitals and nursing homes (MAMTA Friendly Hospitals, MFH) to provide comprehensive maternity services to slum residents. The MFH were paid a low, fixed rate (Rs 4,000) for each institutional delivery by eligible pregnant women (i.e. BPL beneficiaries over 18 years with <2 living children)	A cross-sectional study of beneficiaries and non-beneficiaries was undertaken in six districts of Delhi (33)	210 beneficiaries of MAMTA; 1,77 controls in similar socioeconomic groups	- 23% of eligible non-beneficiaries had a delivery at home last time - 22% of eligible non-beneficiaries had a delivery at private facility last time - 55% of eligible non-beneficiaries had a delivery at a public facility last time	- 30% of beneficiaries had delivery at home last time - 27% of eligible beneficiaries had delivery at private hospital last time - 38% of eligible beneficiaries had delivery at a public facility last time - 5% of eligible beneficiaries had delivery at another type of facility last time
7.9	India	Agra pilot voucher programme paid for safe motherhood, family planning, child immunizations, STI treatment, and outpatient diagnostic services at private facilities for BPL households	Before-after design with no controls, comparing mean population values for voucher service utilization (55)	973 baseline; 505 endline	At baseline, the average use of selected subsidized services: - 26.7% contraceptive prevalence - 30.4% institutional delivery	At endline, the average use of selected subsidized services: - 30.8% contraceptive prevalence - 53.5% institutional delivery Statistical significance not reported
8.10	Kenya	Government of Kenya's output-based voucher programme targets poor women to receive a subsidy either for a long-acting or permanent contraceptive method or a maternal health service package	Before-after design with no controls, comparing mean facility-based deliveries in Nairobi informal settlements before and after launching of the voucher programme. Use of non-voucher child immunization data to falsify alternative hypothesis explaining observed secular increase in RH service utilization (56)	1,927 baseline respondents; 2,448 endline respondents	At baseline, 64.7% of deliveries were at facility (1,238/1,927)	At endline, 71.9% of deliveries were at facility (1,759/2,448) Statistically significant increase in facility-based deliveries with OR=1.4 (95% CI 1.19-1.58)
8.11	Kenya	Government of Kenya's output-based voucher programme targets poor women to receive a subsidy either for a long-acting or permanent contraceptive method or a maternal health service package	A cross-sectional study of a baseline population survey comparing proportion of facility-based deliveries in communities exposed and non-exposed to the OBA voucher programme (17)	887 exposed to vouchers since 2006; 1,191 controls		Among intervention group, 63.4% of deliveries were at facility Among control group, 50.4% of deliveries were at facility Compared to the control group, the intervention group had: - 1.1 (0.8-1.4) non-significant odds ratio of 4+ ANC visits - 1.1 (0.8-1.6) non-significant odds ratio of 1st ANC in 1st trimester - 2.1 (1.5-3.1) significant odds ratio of facility delivery - 2.0 (1.4-2.8) significant odds ratio of skilled delivery care - 1.3 (0.9-1.8) non-significant odds ratio of PNC visit
9.12	Pakistan	In 2010, a one-year pilot voucher programme for maternal health package and reversible short- and long-term contraceptives was implemented in rural Jhang district, Pakistan	A before-after design with controls was used for a difference-in-differences analysis of four RH service utilization outcomes among poor and non-poor quintiles (28)	2,018 mothers interviewed at baseline (1,008 intervention and 1,010 controls); 2,033 mothers interviewed at endline (1,014 intervention and 1,019 controls)	At baseline: - 31% of women in the 4th quintile had facility delivery in intervention areas - 37% of women in the 5th quintile had facility delivery in intervention areas - 25% of women in the 5th quintile had facility delivery in control areas - 31% of women in the 4th quintile had facility delivery in control areas - 7% of women in the 5th quintile received family planning in intervention areas - 8% of women in the 4th quintile received FP in intervention areas - 7% of women in the 5th quintile received FP in control areas - 11% of women in the 4th quintile received FP in control areas	At endline, for facility delivery there was: - 16 ppt increase for the 4th quintile (from 31% to 47%, p<0.01) in intervention areas - 21 ppt increase in facility delivery for the 5th quintile (from 37% to 58%, p<0.001) in intervention areas - 2 ppt increase in facility delivery for the 5th quintile (25% to 27%, NS) in controls - 7 ppt increase in facility delivery for the 4th quintile (31% to 38%, NS) in controls - 3 ppt increase in the use of FP for the 5th quintile in intervention areas (from 7% to 10%, NS) - 4 ppt increase in the use of FP for the 4th quintile in intervention areas (from 8% to 12%, NS) - 2 ppt increase in the use of FP for the 5th quintile in control areas - 1 ppt decrease in the use of FP for the 4th quintile in control areas
9.13	Pakistan	12-month maternal health voucher intervention in southern Punjab, Pakistan, to provide vouchers to low-income households to subsidize maternity care at Greenstar franchised facilities	A before-after design with no controls was used in measuring the change in the mean use of ANC, delivery, and PNC services (27)	681 baseline mothers; 741 endline mothers	At baseline, among pre-voucher women: - 61% had 3+ ANC visits - 61% had facility delivery - 30% had 1 PNC visit	At endline, voucher purchase was crudely associated with: - 22 ppt increase in the use of ANC (61% to 83%, p<0.001) - 19 ppt increase in institutional delivery (61% to 80%, p<0.01) - 31 ppt increase in the use of PNC (30% to 61%, p<0.001)
10.14	Uganda	Voucher programme for maternal healthcare among the poor was implemented by Marie Stopes Uganda in more than 85 private facilities in more than 22 districts in western Uganda 2009-2011	Retrospective difference-in-differences analysis of proportion of facility deliveries as reported in birth history from voucher and non-voucher mothers within 10 km of voucher facilities in western Uganda (57)	175 births in intervention arm before the voucher programme started; 779 births in control arm before the voucher programme started; 434 births in intervention arm after the voucher programme started; 1,184 births in control arm after the voucher programme started	At baseline before voucher programme started: - 30% of future voucher-holders had home delivery - 26% of future voucher-holders delivered in a private facility - 44% of future voucher-holders delivered in a public facility - 38% of non-voucher mothers had home delivery - 18% of non-voucher mothers delivered in a private facility - 43% of non-voucher mothers delivered in a public facility	At endline after voucher programmme ran for more than a year: - 17% of voucher-holders had home delivery - 52% of voucher-holders delivered in a private facility - 30% of voucher-holders delivered in a public facility - 31% of non-voucher mothers had home delivery - 28% of non-voucher mothers delivered in a private facility - 41% of non-voucher mothers delivered in a public facility - 0.6 reduced odds ratio (95% CI 0.3-0.9) of home-birth once programme started for voucher compared to non-voucher mothers - 2.2 greater odds ratio (95% CI 1.3-3.8) of a private facility-based birth once programme started for voucher compared to non-voucher mothers - 0.5 reduced odds ratio (95% CI 0.3-0.9) of a public facility-based birth once programme started for voucher compared to non-voucher mothers
11.15	Uganda	Demand-side voucher and supply-side facility improvements combined to improve access to public, private for-profit and non-profit facilities in two districts of eastern Uganda. Transport for referrals included	Monthly trends in the number of facility-based deliveries reported at voucher and non-voucher facilities from January 2009 to July 2010 (58)	30,000 voucher beneficiaries; Unknown number of controls		Number of institutional deliveries in intervention area more than doubled, ANC and PNC more than tripled while service utilisation in control area remained more or less the same over 18 months of the intervention

† The effect identified depends on the study methodology. For longitudinal data, it represents the change over time in the indicator; and for pre-post comparisons among voucher-exposed groups, it represents the effect of vouchers among the voucher-exposed group compared to the control group;

HEF=Health Equity Fund

SE=Standard error

Most countries where a programme was identified had a Gross National Income (GNI) below US$ 1,400 in 2010 with the exception of Armenia (US$3,200). The GNI of China, Korea, Indonesia, and Taiwan were also higher than US$ 1,400 in 2010 but, at the time of implementation, this was much lower. GNI per capita of low-income countries in 2010 was less than US$ 1,006

lower middle-income countries: US$ 1,006 to US$ 3,975, upper middle-income countries: US$ 3,976-12,275

high-income countries: US$ 12,276 or more (World Bank: http://data.worldbank.org/indicator/NY.GNP.PCAP.CD)

Programme monitoring and evaluation is often limited to tracking utilization of services and vouchers. To some extent, some programmes also track quality indicators (e.g. patients’ satisfaction or providers’ knowledge). Thirteen of the 28 programmes measured technical quality (e.g. clinical care scores or providers’ knowledge), and it is the most commonly-measured aspect of quality in voucher programmes. Nine programmes reported tracking some aspects of clients’ satisfaction.

Fraud control can be built into programme design with multicoloured vouchers and hard-to-counterfeit designs as described in the “Guide to Competitive Voucher Programmes” (24). Seven programmes printed the price on each voucher to reduce the risk that vouchers would be sold above the highly-subsidized price. In programme monitoring, analysts can use programme data to establish a baseline and then look for unusual trends, improbable medical cases, and other suspicious behaviour suggestive of fraud. Thirteen of the 28 programmes had a routine reporting schedule for monitoring purposes. Twelve programmes had information on the type of fraud encountered, ranging from subsidy given to the non-poor, selling voucher above distribution price, fraudulently-issued vouchers and fake Below Poverty Line identity cards, fraudulent claims to informal charges levied at facility (either extra services or double billing), and inadequate service delivery (e.g. missing laboratory services, insufficient consultation time).

## DISCUSSION

### Key findings

This paper assessed key characteristics in 28 maternal health voucher programmes with the goal of identifying characteristics for successful sustainable programmes. The paper intends to contribute evidence for design choices for scaling up maternal health voucher programmes.

### Type of management organization

The most common management organization was a third-party agency tasked to contract accredited facilities and to identify beneficiaries. In addition to contracting providers and delivering subsidies to beneficiaries, voucher management agencies often undertook the core tasks of issuing vouchers, processing claims, accrediting facilities, checking the quality of care, and verifying service delivery. The manifest preference for third-party management could be due to several factors. Efficient claims processing and reimbursement is critical to keeping the providers, particularly private facilities involved in the programme. The programmes that registered significant operational problems with claims and disbursements were either in start-up phase or were public management entities, and, sometimes, existing offices that were repositioned with the added responsibility of managing vouchers on top of other assignments (33,34). In contrast, third-party management agencies can be sanctioned for poor performance and have shown to be responsive to evolving programme needs. In one case, a management agency learned to use multiple communication channels to reach out to beneficiaries and improve the reliability of communications with providers regarding claims reimbursement (35). An important distinction can be drawn between contracted staff in a public institution and contracted third-party organizations.

### Type of service provider

Most maternal voucher programmes contracted healthcare providers from the private sector. Provider mix in a network of contracted facilities reflected the voucher programme's priorities and the policy environment. In spite of perceived high service costs, contracting private providers allowed voucher programmes to extend the reach of social protection services and essentially create additional options for poor healthcare consumers who were previously priced out of the private sector. In two government-managed programmes, private facilities were contracted. However, private providers in the first programme viewed reimbursement rates as too low or payments too slow, and few private facilities remained in the second programme by the end of the initial phase (33,36).

### Provider reimbursement strategy

Performance-based payments can be made to individuals or groups, can reflect service cost or be intended as an incentive, target a few selected services or a wider cluster of indicators, pay incentives for high-quality scores, or pay incentives for treating low-income or disadvantaged clients (37). Likely due to the narrow service package, most voucher programmes make a standard single payment for each service claimed by providers in the network. Usually, single payments are agreed for antenatal care (ANC), normal delivery, delivery with complications, and postnatal care (PNC). In several programmes, tiered pricing distinguishes public from private providers and primary facilities from tertiary hospitals. In programmes with low reimbursement rates, the programme begins to resemble a pay-for-performance mechanism whereby payments are meant to incentivize better care but are not reimbursing for the cost of service delivery at facility as was done in some programmes in India and Bangladesh. Conversely, in Gujarat, a programme paid a market-competitive capitated fee to private providers, which was associated with increased client demand but was not associated with increased treatment of complicated cases (38). The capitated payments would likely reduce claims processing requirements but also reduce incentives to provide expensive care. Disincentivizing complicated care can introduce greater efficiencies but also risks undercutting the quality or adequacy of needed service provision.

### Targeting mechanism

There is significant debate and theory on the extent to which the poor should be targeted for social support rather than creating universal opportunity to access low-cost healthcare. The literature is extensive and by no means conclusive with respect to policy and practice (39,40). Determining whether a universal or targeted policy is socially optimal depends, in a large degree, on the context. In many countries, the challenges with expanding healthcare coverage are threefold: increasing equitable access, improving financial protection, and expanding the quality and size of the benefits package (3). In contexts with highly-inequitable access, the need for subsidies is clustered among the poor, and the cost of a targeting mechanism is more justified. In many low- and middle-income countries, inequitable access to reproductive health services is common (2). Economic poverty is, by no means, the only equity lenses. In Ghana, the National Health Insurance Authority (NHIA) has exempted no fewer than eight disadvantaged groups (youths below 18 years, pregnant women, those aged over 70 years, among others) from paying premiums for national health insurance (41). However, policies for progressive expansion of health coverage would suggest a priority focus on low-income households (42).

Defining the poor can take several forms, including means-testing, proxy means-testing, non-standard (community-based or self-identified) poverty targeting, and geographic targeting of regions with high need (40,43). Furthermore, poverty can be understood as a state of relative deprivation occurring along a continuum from relative plenty to relative scarcity, or it can be seen as a state below an absolute threshold, i.e. a poverty line and calculated as the dollar or local currency equivalent for a basket of consumables.

Aside from the measurement choices to consider in equity targeting, there is the issue of “poverty incidence”. Over any period of time, households may move in to or out of poverty, and there remains a significant practical question of how often to measure poverty in the target population.

Policy-makers are faced with the need to contain operational costs while ensuring high confidence that type I and II errors (e.g. insufficient coverage and subsidy leakage) are kept to a minimum (40). Keeping all else constant, individual targeting is likely to be more costly than geographic targeting or exemptions for readily-identified demographic groups (e.g. pregnant women). If an absolute poverty measurement is used, one may expect to see over the medium to long term that the pool of beneficiaries decreases as the national economy develops.

Beyond the scope of this review is an in-depth discussion of free maternity care or maternity exemptions on user fees and insurance premiums. Suffice to note that performance-based financing programmes without an explicit equity target can improve quality and raise utilization across all wealth quintiles but, as in the case of Rwanda recently, there was no pro-poor, greater-than-average increase in utilization (39). For countries or contexts where a significant gap in equity underscores the need to prioritize poor pregnant women, equity targeting remains an appealing policy option.

From this review, a pattern has emerged in successful programmes, targeting individuals or households through community-based distributors. Even when programmes use geographic targeting, a need remains to mobilize and raise awareness within communities and, in essence, generate demand among beneficiaries who, in the absence of subsidy, would likely have not sought care. Community mobilization appears to be a critical activity.

### Benefits package

Vouchers, unlike insurance, typically offer a narrow benefits package. The voucher itself may have an expiry date, and the range of services for the programmes in this review was largely focused on antenatal care, delivery, and postnatal care. Here again, context came into play and, in some settings, transport subsidies for maternal services were offered.

Providing transport likely increases the administrative cost but increases the likelihood that distributed vouchers will convert into visits to a facility. The programmatic risk in voucher programmes is that great effort was made to distribute the subsidy to beneficiaries who then fail to use the service. Transport subsidies help alleviate the risk of low uptake.

### Unanswered questions and future research priorities

While this taxonomy helps clarify the current state of maternal health voucher programmes and their commonalities, many questions are still unanswered. Aspects of programme cost are still unclear, and the optimal target percentage of administrative costs must be defined. Given the available evidence, a target range should be between 10 and 15%. The challenge is that administrative cost reporting remains non-standard. Setting industry standards would be helpful and would instil greater confidence when comparing administrative efficiency of the programmes.

There is also a need for breakdown of costing information on various aspects, such as marketing strategies, voucher distribution, training of providers, creating and maintaining management information systems, claims processing, administrative and quality monitoring procedures, and fraud detection and control. Additional information in these areas would allow for more robust comparisons of programme implementation. Future research should focus on the cost implications of voucher programme configurations and identify designs that offer optimal allocative efficiency while targeting the bottom economic quintiles. Some specific questions to be addressed include: what is the optimal management to programme cost ratio; can a voucher programme operate on 10-15% management costs and direct the bulk of the services to the bottom two wealth quintiles; is the programme affordable compared to alternatives to achieving the same goals?

Another aspect of voucher programme costs is efficiency, which has also been largely ignored in the prior literature, even though efficient use of resource inputs is often an implicit objective in voucher programmes. One efficiency measure—technical efficiency—is the ratio of resource inputs and service outputs and would be well-suited to reporting in voucher programmes that track financial reimbursements and utilization figures (44-46). Cost-efficiency is particularly relevant when considering the scale of maternal voucher programmes and whether large-scale programmes are feasible and affordable. Maternal health voucher programmes operate in low-resource settings and, in order to be productive, must be able to scale with a relatively small proportion of the government health budget.

This review identified one cost-effectiveness study on maternal health voucher programme (in eastern Uganda) that considered the strategy affordable even under extreme assumptions (47). As a topic for future policy analysis, additional studies are needed from other programmes, taking into consideration their specific conditions. Similarly, it will be a priority to estimate the cost of scaling programmes, taking into account available resources, particularly current government spending on healthcare. In some countries, the National Health Accounts (NHA) reports provide a useful source of information on the level of health expenditure against which the overall cost, technical efficiency, affordability, and ultimately the sustainability of a contemporary voucher programme can be estimated. Future research should examine the technical efficiency of various forms of maternal voucher programmes and focus on the questions such as: if programmes can cost-effectively serve the poor, do clients respond? Is a financial barrier the primary reason for non-use of services before the voucher programme?

While prior studies and reviews have found short-term utilization gains for maternal health voucher programmmes, the long-term health impact and sustained utilization have not been established (Table 3). The current evidence is largely based on very short observation periods, mostly ranging between 3 and 15 months. The review found that the short observation periods to be particularly problematic because a full cycle of comprehensive maternity services from early pregnancy to postnatal care takes nearly a year and a detectable impact on uptake of services, quality and health status would take time for sufficient numbers of users to attend voucher services (15). Future research can examine whether maternal health voucher programmes have long-term health implications, particularly for the women who delivered at facilities because of the programme.

Perhaps, the most substantial unanswered question is whether maternal voucher programme has a population-level health impact. This has not been systematically studied largely because maternal death and disability are relatively rare events, even in a population with high fertility, and sufficient time and strong surveillance is required to detect a significant change in trends (48). Ultimately, the success of large-scale voucher programmes, however, will be judged by their public-health benefit with a measurable reduction in maternal mortality, particularly among women who have delivered at home in the absence of voucher. Perhaps, more than in programmes that monitor resource inputs, voucher managers have an important role to monitor programme's performance in terms of facility-based and community-based maternal death incidence as poor households’ expectations shift to a greater reliance on health facilities to effectively deliver babies that would have previously been delivered at home.

### Limitations of the review

There are several limitations in this review. The search process followed a qualitative approach, which drew heavily from expert knowledge of the field and, as such, was not a systematic review as defined by Cochrane. This review drew from programme reports, expert knowledge, and other sources that were, at times, conflicting. Expert opinion decided which source took precedence. When results of studies were discussed, it could be noted that many studies used weak designs, and none used a randomized design to control for potential unobserved confounders (15). Albeit often weak, the study designs did provide a consistent story with the direction and significance of positive effect as expected for most programmes (Table 3). Such evidence, however weak, is useful. There were frequent comparisons made between voucher programmes, which were useful but care should be taken about how the indicators are constructed and interpreted.

### Conclusions

This review found significant design commonalities in programmes that were functioning at scale. A reliance on contracting out to private providers, employing a third-party management agency, strong community-based mobilization, and individual targeting were common features. There is a surge in the interest and implementation of reproductive health voucher programmes, particularly for maternal healthcare, and this interest creates space for experimentation and learning, if done well. Regional patterns begin to form as neighbouring countries and states take note of local success. As the programmes innovate, there are emerging research needs to address new questions about efficiency, costs, and impact precisely in the market segments where individuals previously did not seek care. A language of common performance metrics and standardized reporting would make comparisons among programmes easier and, as this social protection strategy matures, the research agenda described above will help contribute to a broader understanding of when and where maternal voucher programmes are optimal for reaching those who, in the absence of subsidy, would not have sought care.
